# 233. Efficacy and safety results from the Phase 2 clinical trial of pritelivir versus foscarnet for treatment of acyclovir-refractory and/or resistant mucocutaneous HSV infections in immunocompromised subjects

**DOI:** 10.1093/ofid/ofaf695.085

**Published:** 2026-01-11

**Authors:** Camille N Kotton, Robin K Avery, Kimberly Workowski, Princy N Kumar, Joerg Albrecht, Pranatharthi Chandrasekar, Mayur Ramesh, Anna Wald, Alexander Birkmann, Melanie E Sumner, Bernadette Surujbally, Cynthia Wat, Roy F Chemaly

**Affiliations:** Massachusetts General Hospital, Boston, Massachusetts; Johns Hopkins, Baltimore, Maryland; Department of Medicine, Emory University, Atlanta, GA, USA, Atlanta, Georgia; Georgetown University Medical Center, Washington, District of Columbia; Cook County Health, Chicago, Illinois; wayne state university, detroit, MI; Henry Ford Hospital, Detroit, MI; University of Washington, Seattle, WA; AiCuris Anti-Infective Cures AG, Wuppertal, Nordrhein-Westfalen, Germany; AiCuris Anti-infective Cures AG, Wuppertal, Nordrhein-Westfalen, Germany; AiCuris Anti-Infective Cures AG, Wuppertal, Nordrhein-Westfalen, Germany; Aicuris Anti-infective Cures AG, London, England, United Kingdom; University of Texas MD Anderson Cancer Center, Houston, TX

## Abstract

**Background:**

Treatment of herpes simplex viruses (HSV-1 or HSV-2) mucocutaneous infections in the immunocompromised (IC) host can be difficult due to refractory and/or acyclovir-resistant (ACV R/R) infection. Drug-resistant HSV infection occurs in up to 14% of hematopoietic cell transplant recipients and up to 7% in persons with HIV infection. Foscarnet is the only FDA-approved agent for ACV R/R HSV infection. While effective, foscarnet is associated with significant toxicity including nephrotoxicity and electrolyte disturbances necessitating hospitalization for treatment.

Pritelivir is an investigational novel viral helicase primase inhibitor active against ACV and foscarnet-resistant HSV. Pritelivir showed a statistically significant reduction in viral shedding and clinical lesions in healthy persons with genital HSV-2 infection, compared to valacyclovir. We report the efficacy and safety of oral pritelivir versus foscarnet in a Phase 2 randomized, open label, multi-center comparative trial for the treatment of ACV R/R HSV in IC host; additional subjects received open label pritelivir for foscarnet resistant /intolerance (R/I) infection.

**Methods:**

In Part A, immunocompromised adult subjects with ACV-R/R HSV infection were randomized (2:1) to oral pritelivir (100 mg orally once daily, following a loading dose of 400 mg on Day 1) or IV foscarnet (120mg/kg/day divided every 8 or 12 hrs) for up to 28 days. In Part B, subjects with foscarnet R/I infection received open label pritelivir. Healing rate defined as complete epithelization of lesions, and safety were assessed up to Day 28.

**Results:**

In Part A, 22 subjects were randomized, n=15 to pritelivir and n=7 to foscarnet. Part B enrolled 8 subjects. 40% of patients were immunocompromised due HIV and 47% due to transplant. In Part A, 14 of 15 (93%) in the pritelivir arm and 4 of 7 (57%) in the foscarnet arm healed (95% CI -10,74), (p=0.0766). In Part B, 5 of 8 (62.6%) with foscarnet R/I HSV infection healed. Treatment emergent adverse events leading to drug discontinuation were more common for foscarnet (43%) versus pritelivir (4%).

**Conclusion:**

Pritelivir represents a promising oral anti-HSV agent for the treatment of refractory or resistant HSV infection in immunocompromised patients and is currently in a Phase 3 trial (NCT03073967).

**Disclosures:**

Alexander Birkmann, PhD, AiCuris Anti-Infectives Cures AG: Employee|AiCuris Anti-Infectives Cures AG: Stocks/Bonds (Private Company) Roy F. Chemaly, MD/MPH, ADMA Biologics, Inc.: Advisor/Consultant|AiCuris Anti-Infective Cures AG: Advisor/Consultant|AiCuris Anti-Infective Cures AG: Grant/Research Support|Ansun Biopharma Inc.: Advisor/Consultant|Ansun Biopharma Inc.: Grant/Research Support|Assembly Bioscience: Advisor/Consultant|Astellas Pharma Inc.: Advisor/Consultant|Eurofins-Viracor: Advisor/Consultant|Eurofins-Viracor: Grant/Research Support|Eurofins-Viracor: Honoraria|Gilead Biosciences: Advisor/Consultant|Invivyd, Inc.: Advisor/Consultant|Karius Inc.: Advisor/Consultant|Karius Inc.: Grant/Research Support|Merck and Company, Inc.: Advisor/Consultant|Merck and Company, Inc.: Grant/Research Support|Merck and Company, Inc.: Honoraria|Moderna, Inc.: Advisor/Consultant|Pfizer Pharmaceutc: Advisor/Consultant|Roche/Genentech: Grant/Research Support|SHIONOGI and CO., LTD.: Advisor/Consultant|Takeda Pharmaceutical: Advisor/Consultant|Takeda Pharmaceutical: Grant/Research Support|Tether: Advisor/Consultant

